# Non-Traumatic Unilateral Spontaneous Adrenal Mass Rupture: A Retrospective Case Series Study

**DOI:** 10.3390/medicina62081479

**Published:** 2026-08-01

**Authors:** Hassan Al-Thani, Maryam Al-Sulaiti, Eman Elmenyar, Hussien Touny, Abdelhakem Tabeb, Ayman El-Menyar

**Affiliations:** 1 Department of Surgery, Trauma Surgery, Hamad Medical Corporation, Doha P.O. Box 3050, Qatar; althanih@hotmail.com; 2 Department of Surgery, Hamad General Hospital, Hamad Medical Corporation, Doha P.O. Box 3050, Qatar; malsulaiti4@hamad.qa (M.A.-S.); htouny@hamad.qa (H.T.); atabeb@hamad.qa (A.T.); 3 Faculty of Medicine, Bahçeşehir University, Istanbul 34734, Türkiye; eelmenyar@gmail.com; 4 Department of Trauma Surgery, Clinical Research, Hamad Medical Corporation, Doha P.O. Box 3050, Qatar; 5 Department of Clinical Medicine, Weill Cornell Medical College, Doha P.O. Box 24144, Qatar

**Keywords:** spontaneous rupture, adrenal mass, hemorrhagic, adrenalectomy, IR embolization

## Abstract

*Background and Objectives:* Non-traumatic spontaneous adrenal mass rupture (SAMR) is a rare and life-threatening surgical emergency. It has around a 32% mortality rate based on the underlying pathology. We aimed to explore the presentations, management, and outcomes of SAMR with various pathologies. *Materials and* *Methods*: We reviewed electronic and operative records over the last eight years to identify SAMR at our institute. Patients’ demographics, comorbidities, presentations, pathology, interventions, and outcomes were analyzed. *Results*: There were 14 unilateral SAMR patients who arrived at the emergency department (ED)presenting with abdominal pain. The median age at presentation was 34 years (range 27–56). In total, 12 patients were male (83.3%), and 2 were female, including a 32-week-pregnant woman. Ten were South Asians and four were Arabs. All the patients with SAMR did not have a history of prior adrenal disorders. Nine patients had hypotension at presentation. After the initial clinical examination, the patients underwent a computed tomography (CT) scan in the ED, which revealed unilateral SAMR. There was no medical history of adrenal mass or trauma prior to index admission. The CT scan size of the masses ranged from 5.5 to 23.6 cm (eight were right-sided and six left-sided). Time to intervention ranged from 1 to 11 days. Histopathology showed four pheochromocytomas, four myelolipomas, three normal adrenal tissues with hemorrhagic cysts, one adrenal gland hemorrhage, one pseudocyst, and one angiosarcoma. Six adrenal masses were non-functioning. All patients were managed with open adrenalectomy (two had pre-operative interventional radiological embolization). Blood transfusion was required in most cases. Ten patients required post-operative surgical intensive care with an average stay of 2.42 days. There were no perioperative deaths during the hospital course. *Conclusions*: Ruptured adrenal masses most commonly present with acute abdominal pain requiring urgent surgical intervention, particularly in patients with hemodynamic instability, insufficient initial conservative management or malignancy. Despite the dramatic presentation, outcomes are favorable in experienced centers when early diagnosis and hemodynamic optimization precede interventions and are accompanied by multidisciplinary team involvement.

## 1. Introduction

Non-traumatic spontaneous adrenal mass rupture (SAMR) is defined as the sudden life-threatening, atraumatic rupture of a previously undiagnosed adrenal mass. The precipitating factors hypothesized to trigger SAMR are pregnancy, sepsis, organ failure and post-abdominal surgery. The impact of such stressors causes a rise in catecholamine and adrenocorticotropic hormone (ACTH) levels and a state of platelet hyperaggregability. Consequently, the resultant venous stasis and elevated intraglandular venous pressure cause a rupture of the delicate adrenal venules [1,2,3]. SAMR occurs at an incidence of 0.3% in the general population, underscoring its rarity, and can be fatal if not managed promptly [4]. The reported mortality rates for SAMR vary depending on etiology; pheochromocytoma rupture has a mortality rate of 32% [5], and in cases managed with emergent surgery, the mortality rate could be 45% [1].

Adrenal masses causing SAMR or presenting non-traumatically include pheochromocytoma, adrenal myelolipoma, adrenocortical carcinoma, adenoma, hemangioma, metastases, and pregnancy-related adrenal hyperplasia. Contributing factors for spontaneous adrenal hemorrhage are abundant adrenal vascularity, bleeding disorders, hypercoagulable states, anticoagulant or regular antiplatelet use, infections, and recent surgeries [1,6,7,8,9]. These factors are more often linked with bilateral as opposed to unilateral adrenal mass hemorrhage [9].

The clinical presentation of SAMR is often non-specific, including abdominal pain, hypotension, vomiting, nausea, and fever [8,10]. Therefore, a multidisciplinary acute abdominal diagnostic pathway using a CT scan in the ED is useful for identifying the underlying acute abdominal pathology and shortening the time until intervention [11]. In some cases, adrenal hemorrhage was detected only postmortem due to the initial vague signs and limited imaging [6]. These non-specific symptoms delay prompt diagnosis and management, often until patients are critically ill.

Our case series highlights these challenges and underscores the need for a high clinical index of suspicion. Imaging is essential for diagnosing adrenal hemorrhage, as laboratory tests offer limited guidance. This case series explores the unilateral SAMR with heterogeneous underlying etiologies, all of which shared a similar clinical presentation. In the SAMR, abnormal laboratory values suggesting adrenal insufficiency are rare, unless there is massive bilateral adrenal hemorrhage [6]. Significant changes in hemoglobin and hematocrit typically occur only in these severe instances [8,12]. Therefore, imaging, such as computed tomography (CT), is fundamental for diagnosis and management [8]. We present 14 unilateral SAMR cases with abdominal pain identified by CT in the emergency department. We aim to explore presentation, clinical features, management and outcomes across SAMR etiologies.

## 2. Materials and Methods

This observational, descriptive retrospective single-center case series included 14 SAMR cases at Hamad General Hospital. The inclusion criterion was spontaneous, non-traumatic adrenal gland rupture, regardless of sex, age, or race, occurring between 2017 and 2025. Patients presenting with adrenal mass hemorrhage without evidence of rupture, cases linked to trauma, inadequate imaging or records, or preexisting adrenal disorders that might confound outcomes were excluded. Data was retrospectively collected from institutional electronic health records and operative reports over eight years. Most of our patients underwent Focused Assessment with Sonography for Trauma (FAST) exam in the emergency department (ED); however, the results were inconclusive or non-diagnostic. Rupture of the adrenal gland was primarily identified by abdominal contrast-enhanced CT in the cohort due to patients being hypotensive and hemodynamically unstable. The defining imaging criteria included adjacent perilesional/retroperitoneal hematoma, active contrast extravasation, and/or internal heterogeneity indicative of intratumoral hemorrhage. As the MRI has better sensitivity and is the gold standard for adrenal hemorrhage diagnosis, some patients were transferred from other centers and had an MRI scan to delineate the pathology seen in their CT scans.

Statistical analysis for basic descriptive statistics, such as median, range, percentages, and averages, was conducted whenever applicable. All data analyses were carried out using SPSS version 22 (Armonk, NY, USA). Ethical approval was obtained from the institutional review board (MRC-01-26-012) for the main study protocol. The Medical Research Center (MRC) granted a waiver of consent for publication, provided that no photos or personal identifiers were included.

## 3. Results

This case series included 14 patients (12 males, 2 females) who arrived at the ED presenting with abdominal pain. The median age at presentation was 34 years (range: 27–56). Twelve patients were male (83.3%) and two were female, including a 32-week pregnant woman. Ten were South Asians and four were Arabs. Nine patients had hypotension (systolic blood pressure < 100 mmHg) at presentation. After initial clinical examination, all underwent a computerized tomography scan (CT) at the ED and were found to have unilateral SAMR requiring surgery. There was no medical history of adrenal mass or trauma prior to index admission. Table 1 summarizes the patients’ demographics, comorbidity, investigations, interventions, outcomes and follow-up. Five patients had no comorbidity, four had hypertension, two had diabetes mellitus (DM) and hypertension, one had DM only and one had prior abdominal surgery. The pregnant patient presented with abdominal and back pain, as well as hypotension.

All patients underwent a CT scan, and only three had an additional MRI scan to confirm the diagnosis. The largest adrenal mass found on a CT scan was 23.6 cm (normal adrenal tissue with hemorrhagic cyst), which required a laparotomy for damage control followed by an adrenalectomy. The smallest mass measured 5.5 cm.

Eight patients had right-sided masses and six had left-sided masses. The average time from admission to surgery was 2.8 days. Three patients had delayed surgical intervention (5–11 days) because of unsuccessful initial conservative treatment and either required continuous blood transfusion and continued to have low hemoglobin levels or were transferred from other centers (cases 1, 3 and 5).

All patients underwent open adrenalectomy, and only two patients received interventional angioembolization before surgery. In two patients who underwent angioembolization, the procedure failed to adequately control the bleeding. One patient developed a complication due to the gelfoam retrograde into the renal artery resulting in complete occlusion, which required continuous blood transfusion and eventually an open adrenalectomy. The second patient had active extravasation during the procedure due to other arterial beds that could not be embolized and thus was taken for surgery. Moreover, due to the mass size, there was a high suspicion for malignancy, making angioembolization unsuitable. Also, in the pregnant patients, angioembolization was not offered due to the hazard of radiation.

The pathological findings included four pheochromocytomas, four myelolipomas, three normal adrenal tissues with hemorrhagic cysts, one adrenal gland hemorrhage, one angiosarcoma, and one pseudocyst, with the largest mass measuring 17.5 cm (operative specimen of myelolipoma). In contrast, the smallest mass was a pheochromocytoma measuring 4 cm. All patients had low preoperative hemoglobin levels. The average blood loss during surgery is 1375 mL, and nine patients required transfusion. Complications were reported in three patients: one had wound dehiscence and infection, one underwent embolization in the right renal artery, and one was readmitted at one month for obstructive choledocholithiasis (an irrelevant finding). The average post-operative hospital length of stay was 8 days with no hospital mortality. The ICU stay was dependent on pre- and post-operative findings and as discussed with the anesthesia team. Ten patients required care in the ICU, while four were transferred to high-dependent units (an average stay of 2.42 days). All the patients received the same care from the same surgical and intensive care team.

**Figure 1:**
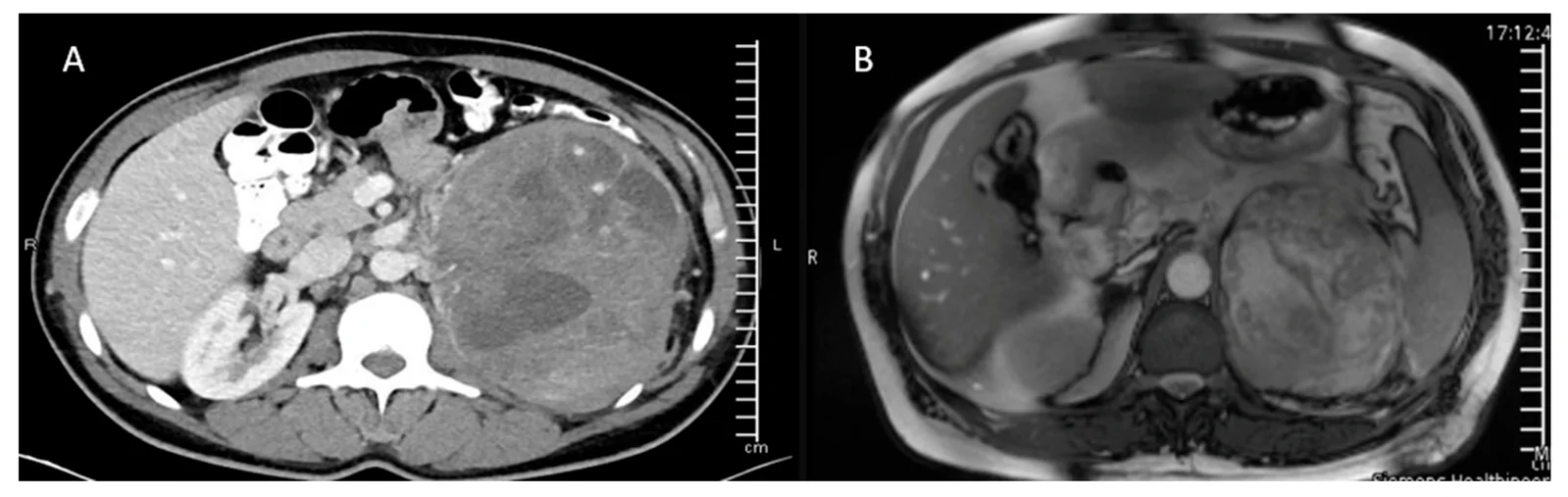
(**A**): CT abdomen: large mass most likely representing a left adrenal carcinoma 16 × 14 cm; however, retroperitoneal sarcoma remains a possibility. (**B**): MRI adrenal with contrast: A 15 cm lesion of the left adrenal gland showing hemorrhagic signal intensity and numerous vessels traversing the lesion (**Case** **1**).

**Figure 2:**
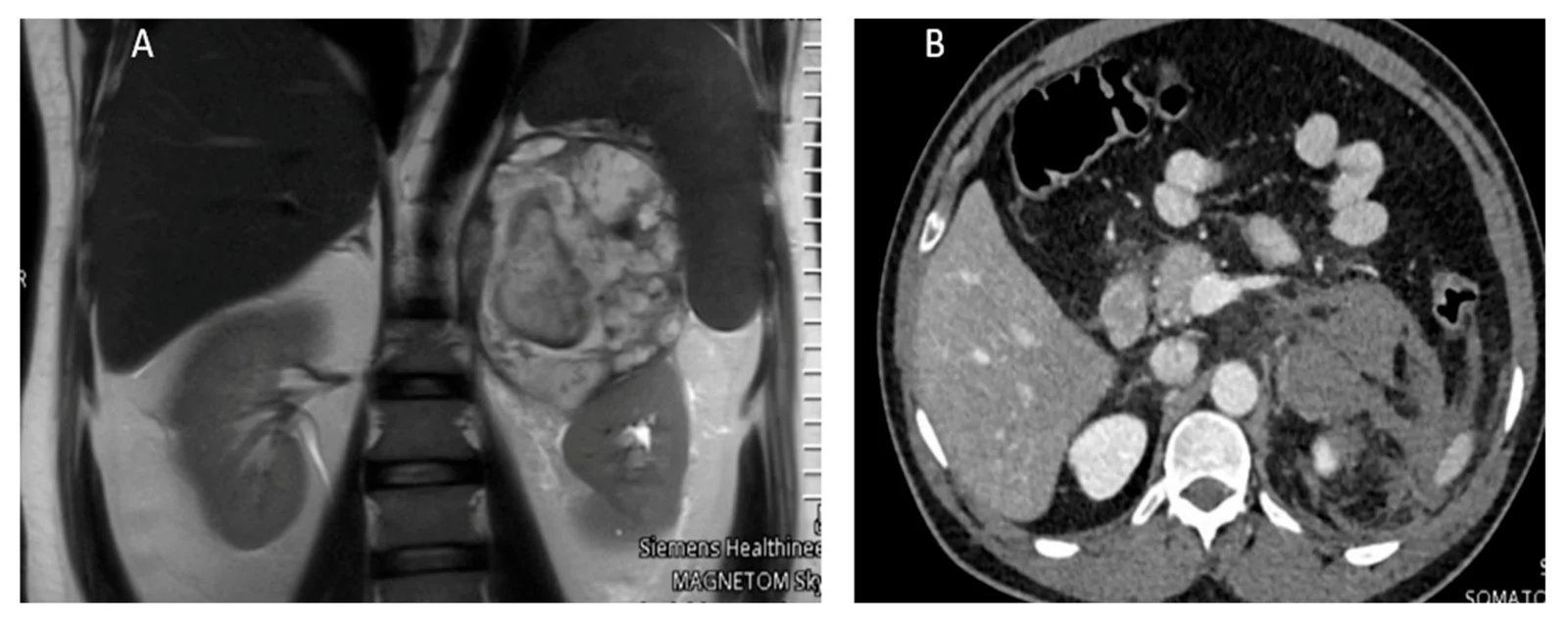
CT scans of abdomen and pelvis with oral and IV contrast plain and at porto-venous phase: (**A**) and (**B**): There is large dense lesion in the left adrenal gland measuring 5 × 4 cm in size with extension and a continuation of a retroperitoneal soft tissue dense lesion, which extends to lower abdomen and measures approximately 14 × 5.5 cm in size, with significant surrounding fat stranding after contrast injection. There is a notable rim enhancement of the remaining peripheral left adrenal gland (**Case 2**).

**Figure 3:**
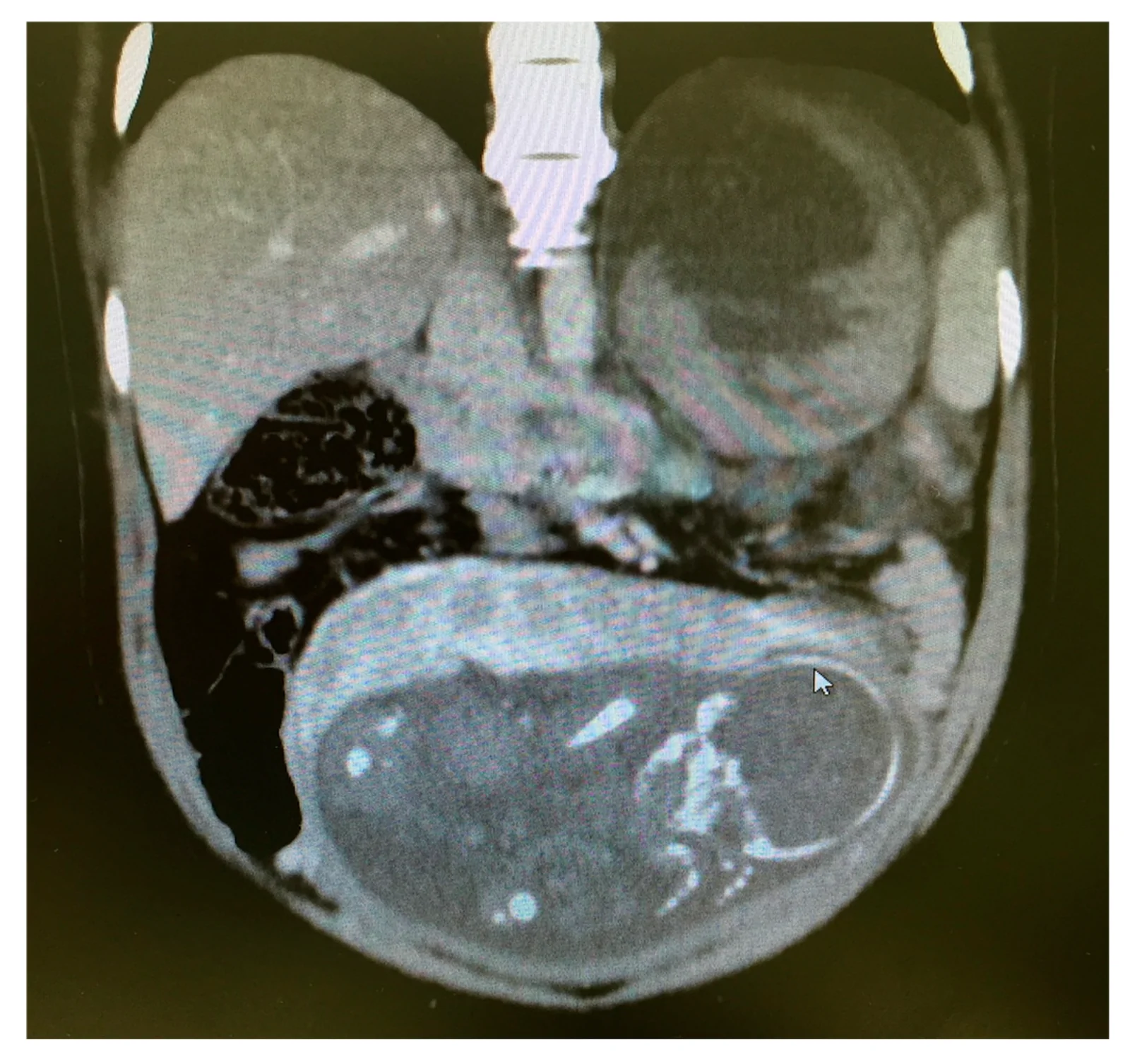
CT scan with IV contrast: A large well-circumscribed hematoma is seen in left suprarenal lesion of the left adrenal, most likely suggestive of left adrenal hemorrhage and a single intrauterine fetus (**Case** **4**).

**Figure 4:**
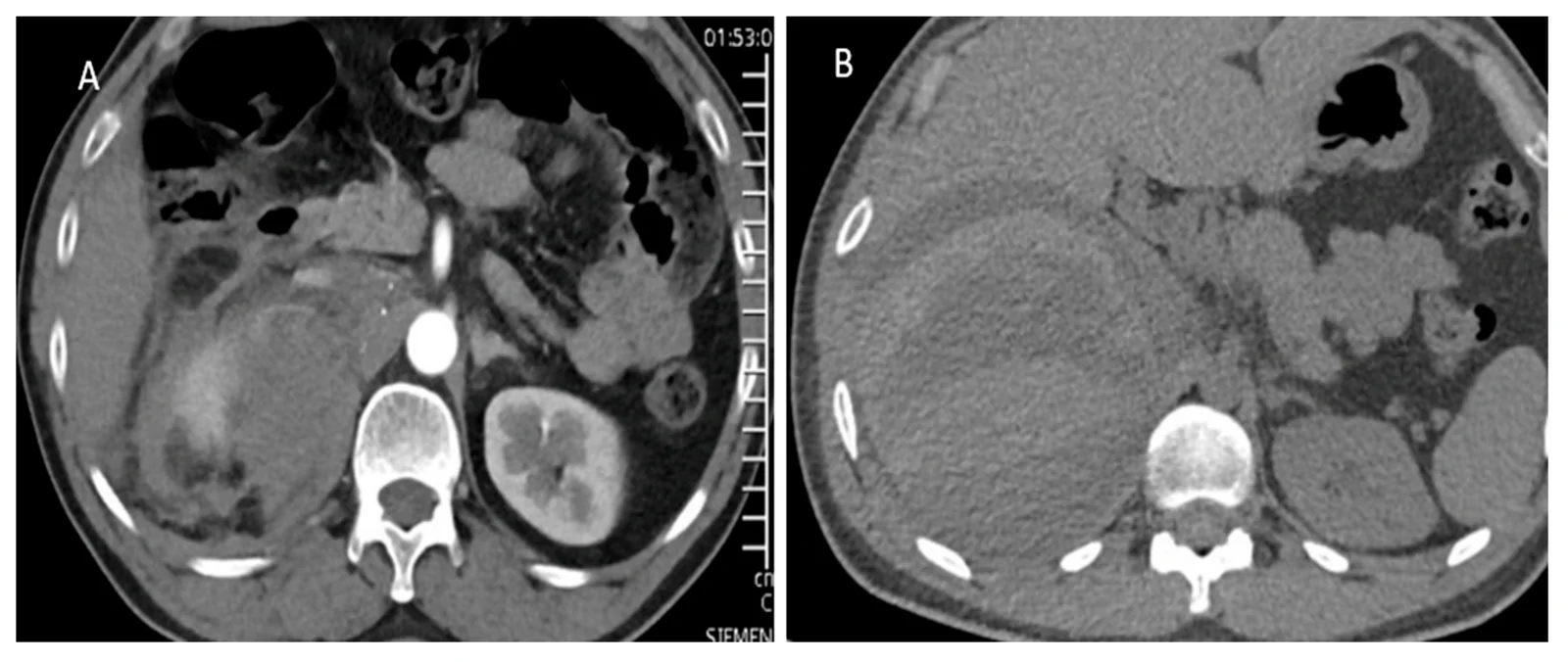
(**A**): CT abdomen with contrast: Enlarged right adrenal gland with an ill-defined peripherally; enhanced right adrenal mass and perilesional/retroperitoneal hematoma extending to the perinephric space (**Case 5**). (**B**): CT urinary tract: A huge heterogeneous collection is seen in the right upper quadrant extending down to the lower abdomen. Measuring 15 × 12 cm, it is heterogenous, indicating a hemorrhagic component (Case 7).

**Figure 5:**
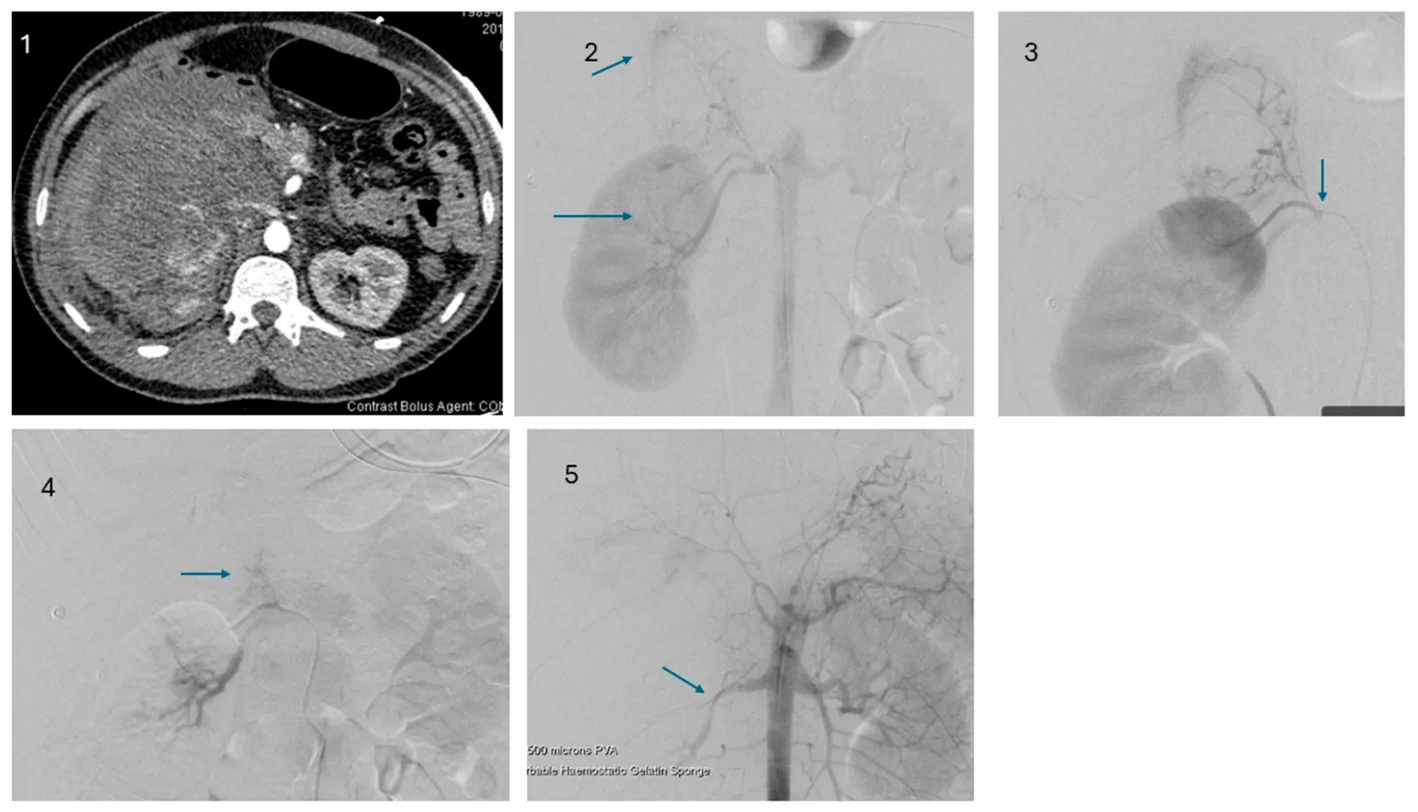
**Case 8: 1.** CT with IV contrast: right hematoma rising from rupture, right adrenal mass with active stabilization of contrast on the arterial phase. **2**–**5.** Angio renal artery: **2** = right adrenal with abnormal arterial supply to the tumor from right adrenal artery. **2** = right renal artery. **3** = selective right adrenal angiogram. **4** = embolized gel foam right adrenal artery. **5** = gelfoam is spilled into the right renal artery with partial occlusion of right renal artery.

**Figure 6:**
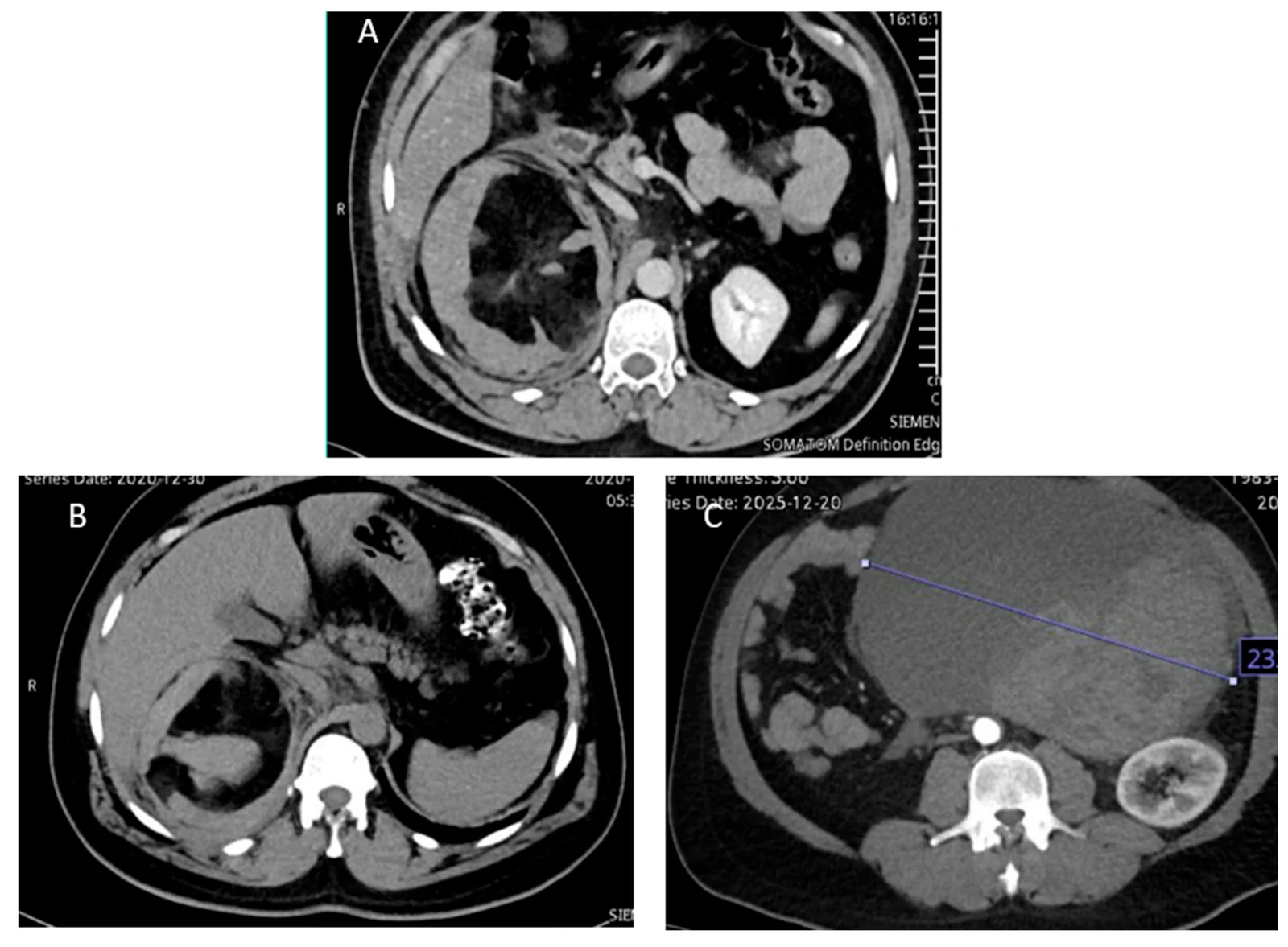
(**A**): CT abdomen with contrast: a large 12 × 11 × 12 cm heterogenous encapsulated round to avoid a mixed-density lesion in the right suprarenal region with mass effect on right kidney, likely representing a large right adrenal myelolipoma complicated by hemorrhage (**Case 11**). (**B**): CT abdomen and pelvis with contrast: Large low attenuation lesion seen in the right suprarenal region, likely originating from the right adrenal gland measuring 10 × 8.5 × 12 cm; features are suggestive of a large right adrenal myelolipoma, with areas of higher density suggesting rupture/hemorrhage (**Case:** **12**). (**C**): Abdomen and pelvis CT with IV contrast: Large intra-abdominal cystic mass lesion with large hematoma showing contrast extravasation on venous and delayed phases only; no arterial extravasation is seen. The patient was transferred from another hospital for surgical intervention (**Case 13**).

Figure 1, Figure 2, Figure 3, Figure 4, Figure 5 and Figure 6 show examples of unilateral SAMR (cases 1, 2, 4, 5, 7, 8, 11, and 13) matching with the serial number in Table 1. Table 2 demonstrates the adrenal mass size, location, pathology, interventions and outcomes of the same order as in Table 1.

## 4. Discussion

Unilateral spontaneous adrenal mass rupture (SAMR) remains an uncommon yet potentially life-threatening clinical entity that poses significant diagnostic and therapeutic challenges, particularly in emergency settings. In this retrospective case series, we evaluated 14 patients presenting with unilateral SAMR arising from diverse etiologies, highlighting the heterogeneity of this condition and the complexity of its clinical presentation. Consistent with prior reports, SAMR often manifests with non-specific and subtle symptoms, which may obscure early recognition. In our cohort, the predominant presenting complaint was abdominal pain of variable onset, localization and severity, a finding that underscores the diagnostic ambiguity associated with this condition and the risk of misattribution to more common abdominal pathologies. However, frontline physicians should have a high index of suspicion in patients presenting with sudden-onset abdominal pain associated with hypotension, particularly in those with a medical history of hypertension. Blomerus et al. proposed a streamlined diagnostic pathway for acute abdominal pain in the ED, including early CT ordering by emergency physicians [11]. Almost three quarters of CT scans revealed clinically significant acute abdominal pathologies, underscoring the diagnostic effectiveness of early CT imaging in this setting. Additionally, CT scan in patients presenting with abdominal pain in the ED, which was the main presentation in our cohort, led to a successful diagnosis in 51% of patients, a 25% increase in confident diagnosis [8,10]. Moreover, CT scan use had a substantial impact on treatment (admission decisions and alterations of the management plan occurred in 25% and 42% of cases, respectively) [13].

The absence of preceding trauma in many cases further complicates the clinical picture and necessitates a high index of suspicion among clinicians. SAMR typically manifests with non-specific signs. The accurate diagnosis and high clinical suspicion are important and can be challenging, particularly when there are no preceding traumatic events. Our cohort is predominantly presented with vague abdominal pain. Recognizing demographic nuances can improve the rapid identification and management of SAMR. According to the latest endocrine society guidelines, early imaging is recommended to reduce the risks associated with delayed diagnosis and management. SAMR is a relatively rare complication of adrenal masses and prompts surgical intervention to prevent a fatal outcome [6]. There are various stressor factors reported to be responsible for precipitating non-traumatic spontaneous adrenal hemorrhage and rupture. These factors include underlying coagulopathies, hypercoagulable states (anti-phospholipid syndrome, systemic lupus erythematosus, and pregnancy), heparin-induced thrombocytopenia following anticoagulant use, antiplatelet use, hypertensive peaks, infections, sepsis (particularly in Waterhouse–Friderichsen syndrome), and recent surgeries [1,6,7,8,9]. Aligning our findings with these standards enhances the credibility and utility of our recommendations for clinicians encountering such atypical cases.

However, most of our reported cases had no pre-existing factors or comorbidities that could lead to SAMR. Moreover, none of our patients had concomitant infections, recent surgeries, known coagulopathies, or were using anticoagulants and/or antiplatelet agents. This high rate of idiopathic cases raises important questions for future research. It would be valuable to conduct prospective studies to explore potential unknown risk factors and mechanisms that may contribute to spontaneous adrenal rupture in patients without known predisposing conditions. Identifying these factors could significantly enhance our understanding of this condition and improve patient management strategies.

It is crucial to highlight the intricate blood supply feeding the adrenal gland, as it makes it susceptible to hemorrhage [1]. The adrenal gland functions like a reservoir sustained by three main inflow arteries, collectively known as the ‘vascular dam’, that drain through a singular outflow vein [1,8]. When stressor factors such as recent surgeries, organ dysfunction, sepsis, or pregnancy occur, they increase catecholamine release, threatening this complex vascular network [1,2,6]. A sudden rise in catecholamines can also elevate intraglandular pressure, potentially leading to rupture, especially if adrenal medullary venous thrombosis is present [1,2]. Furthermore, the adrenal vein’s retention of high epinephrine levels makes it prone to platelet aggregation and vasoconstriction [3].

The most reported etiology for spontaneous adrenal hemorrhage is pheochromocytoma, a catecholamine-secreting tumor with a prevalence rate of 0.1–0.6% in hypertensive individuals and a 5% rate of incidental adrenal tumors [5]. Its spontaneous rupture is uncommon but carries a high mortality rate (one third of cases) [5]; however, there was no mortality in our patients. This zero-mortality outcome clearly contrasts with the literature and underscores the effectiveness of a multidisciplinary approach involving emergency medicine physicians, interventional radiologists, vascular surgeons, and anesthesiologists. The underlying mechanism is the vasoconstrictive effects of elevated catecholamine levels, which further increase intracapsular pressure, leading to tear formation. This, in turn, may cause retroperitoneal hemorrhage and fatal complications if not managed [14]. Likewise, adrenal myelolipoma, which is composed of mature hematopoietic and adipose tissue, carries a high risk of spontaneous adrenal hemorrhage and subsequent hemorrhage [15].

The pregnancy case highlights the need for vigilance in pregnant individuals, since the symptoms can be mistaken for normal pregnancy discomfort, and this may delay diagnosis. Pregnancy has been identified as a precipitant for spontaneous adrenal hemorrhage in between 0.14 and 0.1.1% of patients presenting with hemorrhagic non-functional adenoma [8,16]. The possible pathogenesis is a hypercoagulable state seen in pregnancy, in conjunction with adrenal hyperplasia, resulting in impaired adrenal venous flow [17].

We included one patient who, upon histopathological investigations, was found to have a hemorrhagic ruptured adrenal angiosarcoma, making this the first case in the literature of SAMR of this etiology. Adrenal soft tissue sarcoma is an extremely rare finding that is presented with vague abdominal symptoms, with less than 50 recorded cases worldwide [18].

Our cohort was primarily diagnosed with an adrenal mass through CT modality. CT is more routinely used, particularly in unstable patients, and is sufficient for identifying hemorrhage without requiring a specific adrenal washout protocol [1]. In the ED, a confirmatory CT scan is required to narrow the differential diagnosis list following routine clinical and laboratory tests and prior to therapeutic interventions. However, MRI is the most sensitive and specific diagnostic tool for adrenal hemorrhage [19,20].

**Predictors of adrenal mass rupture and management strategy:** Management of SAMR requires prompt and individualized decision-making, particularly in cases where there is a high suspicion of pheochromocytoma or malignancy, or when the lesion is large. There are several factors that can predict the spontaneous rupture of an adrenal mass, including the tumor type, size, pathology and underlying vascular or coagulation issues. Pheochromocytoma, adrenocortical carcinoma, and large myelolipomas are commonly associated with SAMR [8,21,22]. Definitive treatment in SAMR, particularly as there is a high index of suspicion of pheochromocytoma or a malignancy, as well as if the mass size is more than 6 cm [23]. Other reports showed that tumors (myelolipoma) larger than 6–10 cm or tumor size greater than 10 cm in adults (adrenocortical carcinoma) should be surgically removed [4,24,25]. Angioembolization is recommended as a first-line therapy for SAMR due to pheochromocytoma, followed by surgery, as emergency surgery has a mortality rate of 45% in such cases [24]. Evidently, patients in our cohort who underwent embolization and adrenalectomy were discharged alive. Management strategies ranged from conservative observation and embolization to laparoscopic or open adrenalectomy, guided by hemodynamic stability, capsule integrity, and suspicion of malignancy [4,21,22,24].

Table 3 shows some examples from the literature for adrenal mass with and without rupture including the interventions and outcomes [4,5,16,26,27,28,29,30,31,32,33,34,35]. The predictors of rupture should be identified since presentation, treatment and prognosis are not the same in both conditions (with and without rupture) [5,22,33]. Furthermore, emergency adrenal surgery in the presence of large retroperitoneal hematoma is technically demanding because normal anatomical planes are frequently lost. In these situations, surgery may occasionally require ipsilateral nephrectomy to achieve hemorrhage control or complete resection. However, as our hospital is a referral center with an experienced multidisciplinary team in managing both open and minimally invasive adrenalectomy, none of our patients required nephrectomy despite having spontaneous retroperitoneal hemorrhage. Based on the literature, patients with unilateral SAMR underwent adrenalectomy, including two patients who received angioembolization initially. Only one patient (pregnant) out of the reported ten unilateral SAMR cases, received successful conservative management [16].

**Figure 7:**
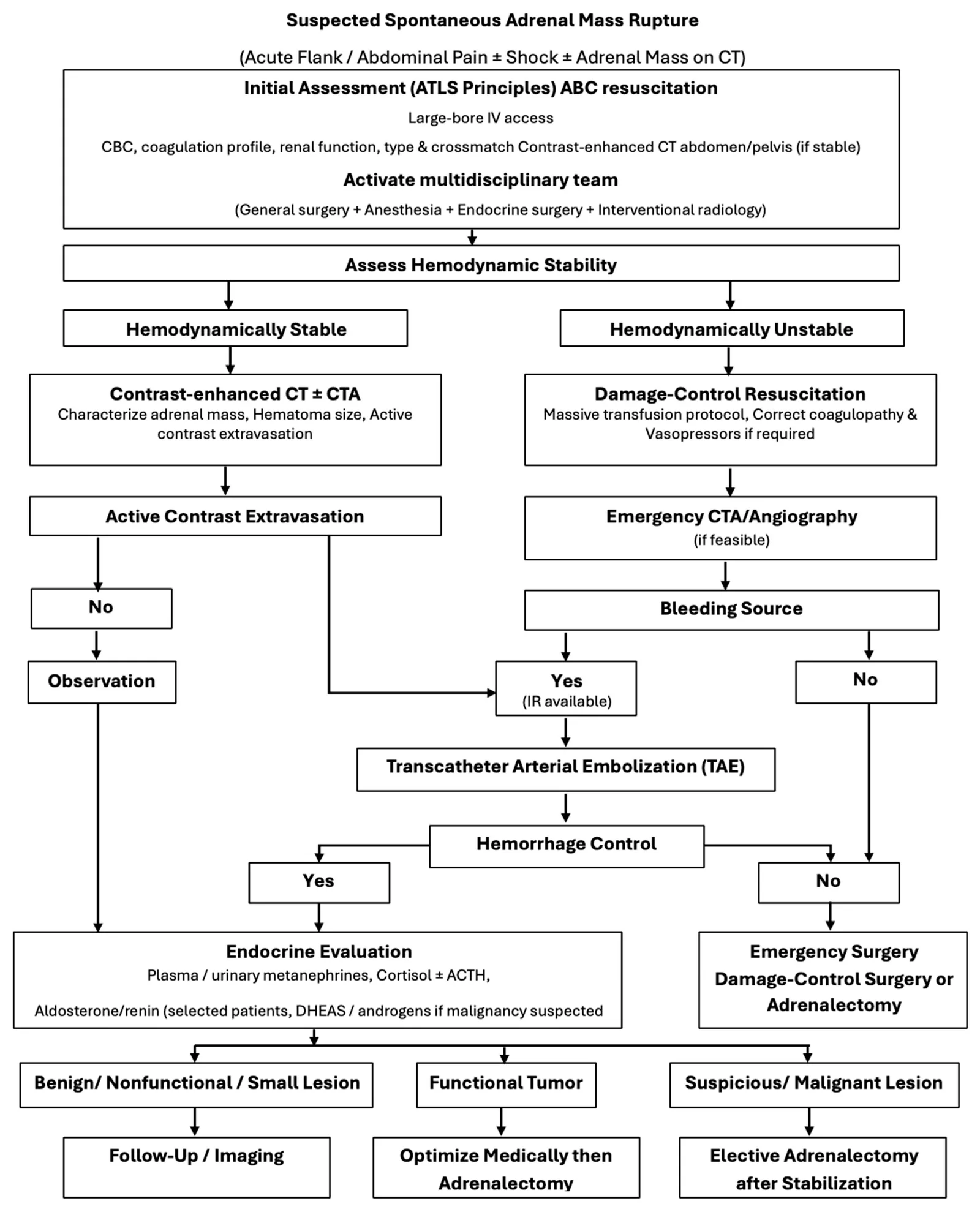
Algorithm for the diagnosis and management of SAMR.

Finally, Figure 7 shows a proposed algorithm for the diagnosis and management of SAMR. This algorithm could be a useful guide for ED physicians after validation.

**Limitations**: The retrospective design and single-center nature of this study are limitations. The sample size is relatively small but remains comparable with recorded numbers in most of the literature (Table 3). Comparison with conservative treatment is not feasible based on the course of this condition. As shown in Table 3, cases from the literature revealed that only a pregnant patient with SAMR, who initially received conservative management, finally underwent surgery.

## 5. Conclusions

SAMR cases are most commonly present with acute abdominal pain and require urgent interventions, particularly in patients with hemodynamic instability, insufficient initial conservative management or malignancy. Despite the dramatic presentation, outcomes are favorable in experienced centers when early diagnosis and hemodynamic optimization precede adrenalectomy, with multidisciplinary team involvement.

## Figures and Tables

**Table 1:** Patient demographics, comorbidity, and interventions.

Case No	Gender (Age)	Comorbidity	Time to Intervention (Days)	Intervention	Hgb (on Admission)	Hgb (Preop)	Blood Transfusion
1	Male (56) Egyptian	Diabetes mellitus	5	Adrenalectomy	8.5	9.6	Yes
2	Male (45) Bangladeshi	Hypertension	1	Adrenalectomy	14.3	12.1	Yes
3	Male (44) Jordanian	Hypertension	5	Adrenalectomy	14	8	Yes
4	Female (28) Indian	Pregnant	1	Adrenalectomy	12	9.5	Yes
5	Male (48) Bangladeshi	Hypertension, Diabetes mellitus	11	Adrenalectomy	13.6	10.7	No
6	Male (30) Tunisia	Hypertension	2	IR and adrenalectomy	12.4	7.9	Yes
7	Male (30) Nepalese	No comorbidity	2	Adrenalectomy	8.5	6.9	Yes
8	Male (27) Nepalese	No comorbidity	1	IR and adrenalectomy	15.3	8.2	Yes
9	Female (37) Indian	No comorbidity	3	Adrenalectomy	10.2	8.3	-
10	Male (37) Bangladeshi	Hypertension, Diabetes mellitus	2	Adrenalectomy	13.6	11.3	Yes
11	Male (46) Bangladeshi	No comorbidity	1	Adrenalectomy	12.8	11.2	No
12	Male (41) Indian	No comorbidity	1	Adrenalectomy	13	10	No
13	Male (42) Jordanian	Sleeve Gastrectomy	1	Damage control laparotomy for bleeding followed by adrenalectomy	11.8	7.4	Yes
14	Male (39) Pakistani	Hypertension	3	Adrenalectomy	14.9	13	No

Abbreviation: Hgb (preop) = blood hemoglobin level (preoperatively).

**Table 2:** The adrenal mass size, location, pathology, and outcomes.

Case Number	CT Scan (Size in cm)	Pathology Size (Size in cm)	Laterality	Length of Hospital Stay	Length of ICU Stay	Pathology	Events	Follow-Up (Days)
1	15	15	Left	16	5	Angiosarcoma		171
2	14	8	Left	3	1	Normal adrenal tissue with hemorrhage		234
3	13	7	Left	14	5	Pheochromocytoma		1559
4	11	10	Right	5	1	Normal adrenal tissue with hemorrhage	wound dehiscence and infection	2426
5	9.6	8	Right	4	0	Pheochromocytoma		927
6	12.6	16	Left	7	6	Pheochromocytoma		2612
7	15	8	Right	7	5	Pseudocyst		101
8	20	4	Right	8	2	Pheochromocytoma		148
9	13.6	5.5	Right	13	0	Myelolipoma	Right renal artery embolization	217
10	12.6	17.5	Left	7	2	Myelolipoma		122
11	12	9	Right	8	2	Myelolipoma		122
12	12.4	14.5	Right	5	0	Myelolipoma		90
13	23.6	15	Left	8	5	Normal adrenal tissue with hemorrhagic cyst	Readmitted after one month with obstructive common bile duct	70
14	5.5	6.5	Right	9	0	Adrenal gland hemorrhage		21

**Table 3:** Examples of unilateral adrenal hemorrhage with and without rupture.

Examples of Unilateral Adrenal Hemorrhage Without Rupture							
Author and Year	No. of Cases	Age/Gender	Presentation	Diagnostic Tool	Diagnosis	Intervention	Outcome
Pushkar et al., 2015 [26]	1	26 years old male	One day left-sided acute abdominal pain	-Contrast-enhanced CT scan showed hemorrhagic collection of 9 × 8.2 × 8.2 cm near the left suprarenal region with 4 × 4.5 × 4.2 cm adrenal mass. MRI: left suprarenal mass with large hemorrhagic component	pheochromoytoma	Angioembolization with Gelfoam Adrenalectomy	Alive
Gavrilova-Jordan et al. 2005 [27]	1	20 years old Primigravid at 33 weeks	Persistent right flank pain Obese	-US abdomen showed a heterogeneous 3.3 × 2.5 × 2.0 cm mass in the right adrenal gland MRI showed a 3.8 × 2.8 × 2.4 cm heterogeneous mass within the right adrenal gland		Conservative management. Mass resolved on CT follow-up 3 months	Alive
Skołozdrzy et al. 2024 [28]	1	32-year-old pregnant woman (32 weeks)	Exacerbation of pain in the right flank	-Low hemoglobin. -MRI revealed a hematoma in the right adrenal gland with dimensions of 12 × 7.3 × 6.7 cm -Control CT following the CS revealed reduction in hematoma		Received 6 units of RBC concentrates and 4 units of FFP. Conservative management until CS.	Alive
**Examples** **of Unilateral adrenal hemorrhage with rupture**							
Chung et al. 2010 [29]	1	36-year-old woman	-Left adrenal mass incidentally detected on abdominal US	-MRI revealed a12 × 10.3 × 9.7 cm sized, heterogeneous mass in the left adrenal gland, with possible internal hemorrhagic necrosis Whole-body positron emission tomography/CT with 18F-fluorodeoxyglucose showed significant 18F-FDG uptake in the huge left adrenal mass	Adrenal cortical carcinoma	Adrenalectomy Replacement and adjuvant chemotherapy	-CT revealed recurrence on 3-month follow-up
Celik et al. 2015 [30]	1	19-year-old male	-Acute chest pain, generalized weakness and mild abdominal pain	-Severe anemia -Elevated cortisol levels -US revealed diffuse free fluid in the peritoneal cavity -CT revealed a perirenal mass and hematoma measuring 10.5 × 12.7 cm.	Adrenal Adenoma	Patient received 6 erythrocyte suspension bags and four bags of FFP A laparotomic left adrenalectomy drainage of the hematoma was performed	Alive
Jou et al. 2013 [5]	1	69-year-old female	-3 days of progressive abdominal pain and fever for 1 day.	-CT revealed 8 × 5 × 4.5 cm^3^ retroperitoneal mass with a hematoma anterior to the left kidney. -Patient became hypertensive with metabolic acidosis and respiratory distress	Pheochromocytoma	Left adrenalectomy during retroperitoneal explorative surgery	Alive
Patel et al. 2022 [16]	1	Primiparous (25 weeks) Female	-25 weeks of gestation presenting with episodes of vomiting, gradually increasing back pain radiating to the abdomen. -Hypertensive	-US revealed a 5 cm right-sided suprarenal mass -MRI confirmed SAH with a hematoma measuring 5.6 × 2.2 cm	-Possible pheochromocytoma hiding within hematoma.	Conservative with successful reduction in mass size on control MRI scan	Alive
Mochizuki et al. 2025 [4]	1	27-year-old male	-Sudden flank pain	-CT revealed a 12 × 11 × 11 cm right retroperitoneal mass with a fat density area and widespread hemorrhage inside the tumor, along with surrounding hematoma -Progressive anemia	Adrenal myelolipoma	-Transcatheter arterial embolization -Laparoscopic adrenalectomy was performed 3 months later	Alive
Christoforides et al. 2013 [31]	1	55-year-old male	-non-tender palpable mass on RLQ on routine follow-up (20 × 20 cm)	CT revealed a huge, well-circumscribed, 23 × 20 cm, cystic lesion. MRI revealed a huge multicystic lesion, in the region of the right adrenal gland.	Idiopathic adrenal hemorrhage	-Exploratory laparotomy; right adrenalectomy	Alive
Bolat et al. 2016 [32]	1	57-year-old female	-acute right flank and RUQ pain with nausea and vomiting -insulin dependent DM	CT revealed a well-defined mass with hypodensities measuring 9.5 × 8.5 cm on the upper pole of right kidney. MRI revealed a 9.0 × 8.7 cm mass on the right adrenal gland localization	Adrenal gland hematoma	-Received fluid and erythrocyte transfusions -Right adrenalectomy	Alive
Zulu et al. 2021 [33]	1	57-year-old male	-one week history of abdominal pain and pleuritic chest pain -Hypertensive	Elevated CRP and renal dysfunction, and anemia and became hemodynamically unstable. US revealed free fluid in abdomen and pelvis with a suprarenal mass measuring 8 × 7 × 3 cm CT revealed d left suprarenal hyperdense mass and retroperitoneal hematoma with perinephric fatty stranding	left adrenal gland infarction and suppurative inflammation	-intra-operative transfusion with 4 RBC units and 2 FFP units -First explorative laparotomy did not identify the source -Second laparotomy with left adrenalectomy was performed	-Died following cardiac arrest
Diaz-Martinez et al. 2026 [34]	1	32-year-old female	sudden, lower back pain	CT scan revealed an adrenal gland of 8.3 × 6.1 × 8.7 cm with heterogeneous density, hypodense, and hypertensive areas of bleeding without enhancement areas (SAH).	Adrenocortical adenoma with cystic degeneration and central hemorrhage	Right adrenalectomy	Survived
Chan et al. 2026 [35]	1	50-year-old male	left-sided flank pain and abdominal distension	CT showed left retroperitoneal hematoma 5.7 × 17 × 8 cm with evidence of active arterial extravasation from a well-defined left adrenal lesion measuring 59 × 53 × 52 mm	7 cm, non-functional adrenocortical carcinoma	Angioembolisation, Laparoscopic left adrenalectomy	

FFP = fresh frozen plasma; RBC = red blood cells; CT = computerized tomography; CRP = C-reactive protein; RUQ = right upper quadrant; RLQ = right lower quadrant; CS = Cesarean section; SAH = Spontaneous adrenal hemorrhage.

## Data Availability

Data will be available upon reasonable request from the primary author after reasonable request and approval from the HMC medical research center.

## Abbreviations

SAMR	Spontaneous adrenal mass rupture
CT	Computerized tomography
MRI	Magnetic Resonance imaging
